# Hiccup-Like Response in a Dog Anesthetized with Isoflurane

**DOI:** 10.1155/2016/8127496

**Published:** 2016-06-15

**Authors:** Enzo Vettorato, Federico Corletto

**Affiliations:** Dick White Referrals, Station Farm, London Road, Six Mile Bottom, Cambridgeshire CB8 0UH, UK

## Abstract

An eight-year-old, female intact Golden Retriever underwent magnetic resonance imaging (MRI) for investigation of urinary and faecal incontinence. Soon after induction of general anesthesia, tracheal intubation, and isoflurane administration, hiccup-like movements were evident. These hiccup-like movements did not respond to hyperventilation and increase of anesthetic. After having ruled out pulmonary disease, the animal was reanesthetized with a similar technique; hiccup-like movements reoccurred and did not stop after discontinuation of isoflurane and commencement of a propofol infusion. Eventually, a nondepolarizing neuromuscular blocking agent was administered to stop the hiccup-like response and allow MRI to be performed. This case report describes the pathophysiology of hiccup-like response and its management in a dog.

## 1. Introduction

Hiccup (or hiccough) is a brief powerful inspiratory effort accompanied by closure of the glottis [1]. In cats, it results from activation of a reflex whose afferent fibres originate from the pharyngeal branch of the glossopharyngeal nerve [2], and the efferent ones from the vagal motoneuron, projecting to the phrenic nerve and laryngeal intrinsic muscles. The central pattern generator of the hiccup reflex seems to be in the reticular formation of the brainstem [3]. Hiccup can occur at any moment of the respiratory cycle, including expiration, but typically follows the inspiratory peak [1].

General anesthesia is described as one of the potential causes of hiccup in medical literature [3–5]. Even if the feline species has been extensively used to study the pathophysiology of hiccup [2, 6–8], no clinical reports are present in veterinary medicine literature describing the* singultus* manifestation in anesthetized animals.

This report describes the hiccup-like response that was encountered in a dog anesthetized with isoflurane.

## 2. Case Presentation

An eight-year-old, female intact Golden Retriever, weighing 27 kg, was referred for investigation of urinary and faecal incontinence, which acutely appeared following two months of chronic vaginal discharge. The latter partially responded to antibiotic treatment. After neurological consultation, the animal was scheduled for magnetic resonance imaging (MRI) of the lumbar-sacral region under general anesthesia.

On preanesthetic examination the dog appeared slightly nervous but in good physical condition (ASA II). The heart rate was 90 beats* per* minute (bpm), respiratory rate was 15 breaths* per* minute (brpm), pulse quality was good, mucous membranes were pink, and capillary refill time was less than 2 seconds. Thoracic and cardiac auscultation were unremarkable, as were the results of hematological and biochemical blood tests.

After a mild sedation was achieved administering methadone intramuscularly (0.2 mg kg^−1^; Synastone, Auden Mckenzie Ltd., UK), anesthesia was induced intravenously 30 minutes later with fentanyl (3 *μ*g kg^−1^; Sublimaze, Janssen-Cilag Ltd., UK) and propofol (2.5 mg kg^−1^; Rapinovet, Schering-plough Animal Health UK). The trachea was intubated with a cuffed tube; the cuff was inflated; intubation was unremarkable. Anesthesia was maintained with isoflurane (IsoFlo, Abbott Laboratoires, UK) in 100% oxygen, delivered through a circle system. Monitoring consisted of capnography, measurement of inspired and expired anesthetic gases and oxygen, measurement of noninvasive arterial blood pressure (Datex AS3, Helsinki, Finland), and esophageal stethoscope.

Shortly after commencement of isoflurane administration, a gasping breathing pattern was noted, with a respiratory rate of 40–50 brpm and jerk movements of the mouth and all four limbs. Heart rate was 140 bpm. Inadequate depth of anesthesia was considered the cause of the observed movements; thus ventilation was assisted manually (15 brpm) and the vaporizer setting was increased from 2% to 3% (oxygen 3 L min^−1^) in order to deepen the anesthetic plane. Further, a bolus of fentanyl (1 *μ*g kg^−1^) was administered intravenously. At that time, end-expiratory carbon dioxide tension (PE′CO_2_) was 22 mmHg. Because the respiratory pattern did not change during the following 10 minutes, and suspecting an underlying pulmonary disease, MRI was cancelled while thoracic radiographs and an arterial blood gases analysis were performed. Radiographs were unremarkable and alkalemia due to primary respiratory alkalosis was apparent in the arterial blood gases analysis results (Table 1). The dog was allowed to recover from general anesthesia. Once the vaporizer was turned off and the breathing system flushed with oxygen, the dog's breathing pattern improved and became normal. The recovery from general anesthesia was uneventful. MRI was rescheduled for the following day.

On day 2, preanesthetic assessment was unremarkable and similar to that obtained the previous day. Dexmedetomidine (1.25 *µ*g kg^−1^ Dexdomitor, Orion Pharma, Finland) and methadone (0.25 mg kg^−1^) were administered slowly intravenously. The resulting sedative effect was good with the animal relaxed in lateral recumbency. Anesthesia was induced with propofol (1.5 mg kg^−1^) and, after intubation of the trachea with a cuffed tube, maintained with isoflurane (vaporizer setting was 3%) in 100% oxygen at the flow of 3 L min^−1^, delivered through a circle system. Also in this occasion tracheal intubation was unremarkable.

As on day 1, the animal started gasping and jerking continuously as soon it was connected to the breathing system. At that point, isoflurane was immediately turned off and the breathing system flushed with pure oxygen. Anesthesia was then maintained with a constant rate infusion (CRI) of propofol (0.3 mg kg^−1^ min^−1^), after slow administration of a loading dose (0.5 mg kg^−1^). As the animal's breathing pattern did not improve, atracurium (0.2 mg kg^−1^, Tracrium Injection, GlaxoSmithKline, UK) was administered intravenously and intermittent positive pressure ventilation (Penlon Nuffield 200 ventilator) was started to maintain eucapnia (PE′CO_2_ 35–45 mmHg). Respiratory rate was set to 15 breathes per minute, tidal volume was 300 mL, and peak inspiratory pressure was 12 cmH_2_O. The rest of the anesthetic time was uneventful, but it was necessary to top up atracurium every 15–20 minutes, because hiccups restarted as soon as neuromuscular function started returning.

An extensive invasive sacrococcygeal neoplasia was found on MRI. The owner decided to euthanize the dog but declined postmortem examination.

## 3. Discussion

In contrast to eupneic breathing, hiccups combine a sudden powerful coordinated burst of the inspiratory muscles of the thorax, diaphragm, neck, accessory, and external intercostal muscles with an inhibition of the expiratory abdominal muscles, active movement of the tongue toward the roof of the mouth, and active adduction of the glottis, which occurs after the beginning of inspiratory flow and it is responsible for the peculiar sound [1]. Differently from sneezing and coughing, hiccup is not a protective reflex. In fact, during coughing and sneezing a vigorous expiratory effort is produced causing expulsion of irritants in the upper respiratory airway [9]. Moreover, hiccup has to be distinguished from reverse sneezing. The latter is a mechanosensitive aspiration reflex and consists of a paroxysmal inspiratory effort (stertor) associated with adduction of laryngeal cartilages. The negative pleural and tracheal pressure generated by the inspiration allows an increase of the inspiratory inflow once the glottis opens, resulting in potential aspiration of foreign material trapped in the nasopharynx [9].

The physiology of hiccup has been extensively studied in cats [2, 6–8]. The mechanical (cotton-tipped swab) stimulation of the dorsal aspect of the epipharynx has been associated with hiccup. The response to such stimulation is characterized by a strong inspiratory effort, large peak of negative inspiratory pressure (less then −20 cmH_2_O), spasmodic activity of the diaphragm, no contraction of the abdominal muscles, and phasic inhibition of the abductor laryngeal muscle (posterior cricoarytenoid muscle) [7]. In particular, the electrical stimulation of the pharyngeal branch of the glossopharyngeal nerve (PB-IX) is responsible for hiccup. On the contrary, the stimulation of the main trunk of the IX cranial nerve evokes an expiratory reflex (cough), but not an inspiratory (hiccup-like) response [2]. The lower brainstem, and more precisely the reticular formation, seems to be the area where the central connection of the hiccup-like reflex is located [6]. Interestingly, the coordinating motor pattern of coughing and sneezing has been occasionally elicited by an electrical stimulation applied to area in proximity to those generating hiccup [6]. Even if PB-IX was not directly stimulated in the dog of the present report, we believe that the breathing pattern seen can be described as a hiccup-like response.

A light plane of anesthesia was initially suspected to be the reason of the abnormal breathing pattern. Fentanyl was administered as a respiratory depressant and manual IPPV was started in order to increase the alveolar ventilation and therefore the uptake of anesthetic agent. Further, the lung's inflation, activating the stretch receptors in the bronchi and bronchioles, can stimulate the Hering-Breuer reflex, which should inhibit the normal respiratory pattern and should also inhibit hiccup [10]. According to Butt Jr. et al. [3], hiccupping during anesthesia can be managed by deepening anesthesia and hyperventilation with the aim of causing apnoea and then inhibition of spontaneous ventilation. Moreover, continuous positive airway pressure seems to be an efficacious method to stop hiccup during anesthesia [11]. However, in the presence of hiccup, the decrease of arterial carbon dioxide tension (PaCO_2_) caused by IPPV can produce the opposite effect. The frequency of hiccups seems to be inversely correlated to the PaCO_2_: the frequency of hiccup decreases with increasing PaCO_2_ [1], and this may be the physiological explanation for the old notion that breath-holding will stop hiccups. This could also explain why the dog did not respond to our initial treatment, which resulted in hyperventilation and respiratory alkalosis, as confirmed by arterial blood gas analysis. While in children in whom the trachea was not intubated hiccup was associated with a decrease in respiratory frequency and minute volume, oxygen desaturation, and relative bradycardia, hiccupping spells were characterized by hyperventilation and respiratory alkalosis if the trachea was intubated [12].

The lack of pulmonary disease, ruled out by thoracic radiographs, the acute onset of hiccup following commencement of isoflurane administration, and establishment of a normal breathing pattern as soon as the animal was disconnected form the breathing system were the reasons for suspecting that the hiccup was an unusual adverse reaction of the animal to the volatile anesthetic. Activation of *γ*-aminobutyric acid (GABA)_A_ receptors may facilitate hiccup in humans; on the contrary, baclofen, a GABA_B_-receptor agonist, is one of the most effective drugs for the treatment of intractable hiccup [13]. The interaction between halogenated anesthetics and GABA receptors is one of the possible mechanisms of action by which volatile anesthetics depress the central nervous system (CNS) and produce unconsciousness [14]. Isoflurane facilitates the hiccup-like reflex in cats by activating the central and peripheral GABA_A_-receptors, but it also suppresses it by activating central and peripheral GABA_B_-receptors [8]. However, the same study showed that the hiccup-like response is inhibited proportionally to the alveolar isoflurane concentration, thus lending support to Butt's theory [3]. In the case here reported, hiccup did not respond to hyperventilation and deepening of anesthesia, and for this reason a constant rate infusion of propofol was started. Persistence of hiccup during propofol infusion may be explained by the fact that propofol, as well as benzodiazepines and ultrashort acting barbiturates, may facilitate hiccup in humans by activating GABA_A_ receptors [15–17].

Intubation, with consequent stimulation of the glottis seems to be a possible cause of hiccup in humans [18]. The use of supra glottis airways devices (laryngeal mask, LMA) has been advocated as a possible treatment for hiccup [19], even if 74/179 cases of hiccup after induction of anesthesia were trigged by LMA insertion [20]. Unfortunately, a LMA was not available at the time. Total intravenous anesthesia without endotracheal intubation or any other mean of securing the airway could have potentially stopped the hiccupping episode; however as hiccup has been associated with relaxation of the lower oesophageal sphincter [21, 22] with reflux occurring in up to 40% of human beings with hiccup [23], this would not be a safe option.

Several drugs and techniques have been considered for treatment of hiccup in humans [3–5, 24]. Ketamine (0.4–0.5 mg kg^−1^ IV) has been reported to rapidly terminate hiccup during anesthesia [25] and in the postoperative period [26] by acting centrally as well as at the spinal cord level [27]. Even if the hiccup did not stop by deepening anesthesia we cannot exclude that a more profound preanesthetic sedation would have helped in controlling such hiccup-response.

Neuromuscular blocking (NMB) agents are probably the most reliable way to stop hiccup during anesthesia, rapidly stopping diaphragmatic contractions. However, they cannot be considered as specific treatment of hiccup, because they do not remove the real cause of the* singultus* and do not affect the neuronal circuitry that produces it. In fact, when the paralysis starts to wear off, the return of the diaphragmatic activity may be associated with a new hiccup-like pattern, as in the case here reported. Furthermore, the use of NMB agents has to be judicious: if inadequate plane of anesthesia or surgical stimulation is suspected as cause of hiccup, the use of NMB agents should be avoided, and anesthesia should be deepened and/or analgesics should be administered prior to consideration of neuromuscular block.

## 4. Conclusion

Acute hiccup is a potential complication that can occur during sedation and general anesthesia not only in humans but also in dogs. Many interventions have been proposed to treat it, but the current literature does not suggest a single effective treatment. The administration of NMB agents can be helpful when a real cause of hiccup cannot be identified and inadequate anesthesia and analgesia have been ruled out as possible causes.

## Figures and Tables

**Table 1:** Arterial blood gas collected taken from a dog with hiccup-like response while anesthetized with isoflurane in oxygen 100%. PaCO_2_: arterial carbon dioxide partial pressure; PE′CO_2_: end-expiratory partial pressure of carbon dioxide; PaO_2_: arterial oxygen partial pressure; FiO_2_: inspiratory fraction of oxygen; HCO_3_^−^: bicarbonate; Na^+^: sodium; K^+^: potassium; Cl^−^: chloride.

pH	PaCO_2_	PE′CO_2_	PaO_2_	FiO_2_	HCO_3_^−^	Na^+^	K^+^	Cl^−^
	mmHg	mmHg	mmHg	%	mmol L^−1^	mmol L^−1^	mmol L^−1^	mmol L^−1^
7.49	29	22	541	89	18.7	157	3.2	118
